# The Widespread Presence of a Multidrug-Resistant *Escherichia coli* ST131 Clade among Community-Associated and Hospitalized Patients

**DOI:** 10.1371/journal.pone.0150420

**Published:** 2016-03-01

**Authors:** P. Martijn den Reijer, Sebastian van Burgh, Arjan Burggraaf, Jacobus M. Ossewaarde, Anneke van der Zee

**Affiliations:** 1 Department of Medical Microbiology, Maasstad Hospital, Rotterdam, The Netherlands; 2 Department of Medical Microbiology and Infectious Diseases, Erasmus Medical Centre Rotterdam, The Netherlands; St. Petersburg Pasteur Institute, RUSSIAN FEDERATION

## Abstract

**Background & Aims:**

The extent of entry of multidrug-resistant *Escherichia coli* from the community into the hospital and subsequent clonal spread amongst patients is unclear. To investigate the extent and direction of clonal spread of these bacteria within a large teaching hospital, we prospectively genotyped multidrug-resistant *E*. *coli* obtained from community- and hospital associated patient groups and compared the distribution of diverse genetic markers.

**Methods:**

A total of 222 *E*. *coli*, classified as multi-drug resistant according to national guidelines, were retrieved from both screening (n = 184) and non-screening clinical cultures (n = 38) from outpatients and patients hospitalized for various periods. All isolates were routinely genotyped using an amplified fragment length polymorphism (AFLP) assay and real-time PCR for CTX-M genes. Multi-locus sequence typing was additionally performed to confirm clusters. Based on demographics, patients were categorized into two groups: patients that were not hospitalized or less than 72 hours at time of strain isolation (group I) and patients that were hospitalized for at least 72 hours (group II).

**Results:**

Genotyping showed that most multi-drug resistant *E*. *coli* either had unique AFLP profiles or grouped in small clusters of maximally 8 isolates. We identified one large ST131 clade comprising 31% of all isolates, containing several AFLP clusters with similar profiles. Although different AFLP clusters were found in the two patient groups, overall genetic heterogeneity was similar (35% vs 28% of isolates containing unique AFLP profiles, respectively). In addition, similar distributions of CTX-M groups, including CTX-M 15 (40% and 44% of isolates in group I and II, respectively) and ST131 (32% and 30% of isolates, respectively) were found.

**Conclusion:**

We conclude that multi-drug resistant *E*. *coli* from the CTX-M 15 associated lineage ST131 are widespread amongst both community- and hospital associated patient groups, with similar genetic diversity and similar distributions of genetic markers.

## Introduction

Multidrug-resistant *Enterobacteriaceae*, including *Escherichia coli*, are detected at an increasingly alarming rate throughout the world [1–3]. The increasing resistance of *E*. *coli* is mainly attributed to the pandemic spread of plasmids harbouring CTX-M cefotaximases, conferring resistance to both beta-lactam antibiotics and other antibiotic groups, including fluoroquinolones and aminoglycosides [4–7]. In addition, many multi-drug resistant *E*. *coli* are also associated with significant pathogenic potential, causing a wide range of infections such as the isolates belonging to the emerging ST131 clonal complex [8]. Together with a rapidly declining pipeline of new antibiotics, this emergence of multi-drug resistant, pathogenic *E*. *coli* raises serious concerns about patient and public health [9–11].

In this context, European and national guidelines [12] now recommend to screen for multi-drug resistant micro-organisms (MDROs) including *E*. *coli*. Although screening has been demonstrated to effectively control hospital-associated outbreaks [13–15], the optimal strategy for control of multi-drug resistant *Enterobacteriaceae* in non-outbreak settings remains under debate [3, 16–19].

One important factor that might hamper the effectiveness of screening and transmission control of multi-drug resistant *Enterobacteriaceae* is their apparent different molecular epidemiology compared to other MDROs. While MDROs such as vancomycin-resistant *Enterococci* [20, 21] and carbapenem-resistant *Pseudomonas* [22–24] are typically associated with hospital outbreaks, *Enterobacteriaceae* appear to be mainly community acquired and enter the hospital from the community [25]. For instance, it was recently demonstrated that almost half of all extended spectrum beta-lactamase (ESBL) producing *E*. *coli* were already detected in patients at admission [3] and both the prevalent CTX-M 15 and the ST131 clonal complex are widely detected in both the hospital and community [4–8]. However, the extent of clonal spread of multi-drug resistant *E*. *coli*, and the extent to which this pathogen is acquired within the community or in the hospital remains unclear. More insight into these matters is essential to optimize strategies for management and control of this serious threat to public health.

Active screening for MDROs, including multi-drug resistant *E*. *coli*, has been recently implemented by our laboratory following several outbreaks with MDROs in our and other Dutch hospitals [26–28]. In agreement with current national guidelines, all *E*. *coli* identified in our laboratory that are resistant to multiple antibiotic groups and/ or are confirmed to produce ESBL are labelled as MDRO and appropriate transmission control measures are undertaken. In addition, various patient groups at high risk of acquiring MDROs, such as intensive care unit patients and patients hospitalized for more than one week, are actively screened for MDROs using both selective cultures and rapid molecular screening [12, 29].

In this study, multi-drug resistant *E*. *coli* identified in our laboratory were genotyped using an in-house amplified fragment length polymorphism (AFLP) assay, which has a high discriminatory power as previously validated in outbreaks with other *Enterobacteriaceae [30, 31].* Results were confirmed using real-time PCR for CTX-M groups and multi-locus sequence typing (MLST). Our aims were to investigate the extent and direction of clonal spread of these organisms amongst our patients. Genotyping results were compared between patients that were more likely to carry community-associated isolates and those that were more likely to have acquired hospital-associated isolates.

## Material and Methods

### Ethics statement

All bacterial isolates used in this study were obtained from cultures that were taken in the context of routine patient surveillance, in accordance with national guidelines [12], or in the context of routine diagnostics. Since no extra action or sampling was requested other than the medically intended and only patients were included that did not opt-out for further use of cultures for scientific research, written informed consent was not obtained and no ethical approval was required in conformity to the codes of conduct for responsible use and health research, formulated in agreement with national law by the Federation of Dutch Medical Scientific Societies (Federa) [32] [33]. PMdR coded all bacterial isolates and basic demographic data of patients and only qualified physicians of the department of Medical Microbiology (PMdR and JMO) had access to the original patient data. This procedure was approved and the acquisition of additional written consent was waived specifically for this study by the Medical Ethics Committee of the Maasstad Hospital Rotterdam.

### Patients

During the study period from April 1^st^ until September 1^th^ 2013, the first isolated multi-drug resistant *Escherichia coli* from 222 patients was included in this study. For all patients basic parameters including age, gender and postal zone were recorded. In addition, the number of hospitalizations during the previous year, the hospitalization status and location at time of strain isolation was documented. The number of days between admittance and strain isolation for all hospitalized patients was documented. Based on this, patients were divided into two groups: group I patients were either not hospitalized or hospitalized less than 72 hours at time of strain isolation while group II patients were hospitalized for at least 72 hours at time of strain isolation. The cut-off of 72 hours was chosen in line with other recent studies [3, 17] as not to overestimate the number of hospital-acquired isolates [18, 34].

### Cultures

In agreement with national guidelines [12], diverse patient groups in our hospital at high risk of acquiring multi-drug resistant micro-organisms (MDROs), such as patients admitted to the intensive-care unit and patients hospitalized for at least one week irrespective of ward, were screened weekly with throat- and rectal swabs for MDRO carriage. In addition, some patient groups at high risk of MDRO carriage, including patients with hospitalization in the past 6 months abroad, were screened once at admittance or in the outpatient setting. MDROs in all these screening cultures were identified using both rapid molecular screening [12, 29] and incubation in selective broth (see section ‘Bacterial isolates‘ below).

In addition to isolates obtained from screening cultures, all isolates from (non-screening) clinical cultures identified as multi-drug resistant based on routine susceptibility testing using the VITEK® 2 system (bioMérieux) were also included in this study. The majority of 184 out of 222 isolates (83%) was obtained from screening cultures. These screening cultures were mainly rectal swabs, with the exception of 7 throat cultures, 7 urine cultures and 3 drain cultures. The remaining 38 (17%) non-screening, clinical cultures containing multi-drug resistant (MDR) *E*. *coli* were mainly obtained from urine, with the exception of three wound cultures, one broncheo-alveolar lavage and one blood culture.

### Bacterial isolates

MDR isolates from screening cultures were obtained after incubation of swab material in selective broth containing ceftazidime, cefotaxime, and the combinations gentamycin/ ciprofloxacin and tobramycin/ ciprofloxacin. Isolates growing in these broths were plated on ESBL CHROMagar™ or gentamycin/ ciprofloxacin or tobramycin/ ciprofloxacin plates, respectively. Antimicrobial resistance and ESBL production of isolates growing on these plates were confirmed with respectively VITEK® 2 susceptibility testing and disk diffusion assays. Multi-drug resistance of *E*. *coli* was defined as resistance to aminoglycosides (gentamycin and/or tobramycin) and quinolones (ciprofloxacin) and/or extended-spectrum beta-lactamase (ESBL) production confirmed with disk diffusion assays [12] (Oxoid, Thermo Scientific).

MDR isolates from (non-screening) clinical cultures were directly identified based on VITEK® 2 susceptibility testing. Additional disk diffusion assays to confirm ESBL production were performed in case of resistance to ceftazidime and cefotaxime. Antimicrobial susceptibility for *E*. *coli* in the VITEK® 2 system was determined using card AST-N199 (bioMérieux).

### Genotyping of MDR *E*. *coli*

All MDR *E*. *coli* were genotyped using an in-house amplified fragment length polymorphism assay, as previously described [31]. In short, DNA was extracted from isolates using the Sigma GenElute™ Bacterial genomic DNA kit (Sigma-Aldrich Co.) according to the manufacturer’s protocol. The restriction-ligation reaction was performed with enzymes *Eco*RI, *Pst*I, *Xba*I, and *Nhe*I (Roche Molecular Biochemicals) and adaptor oligonucleotides for 2–3 hours at 37 ^0^C. DNA fragments were precipitated and then amplified with primers complementary to the adaptors ligated to EcoRI and NheI sites; peco-T and pn. These primers were chosen based on the resolution of banding patterns produced by this primer combination for *E*. *coli* strains (data not shown). Banding patterns were visualized by agarose gel electrophoresis and gel images were made using the Auto Chemi Imaging System (UVP, LLC). Images were analyzed using Bionumerics software version 7 (Applied Maths, Sint-Martens-Latem, Belgium). Banding patterns were compared using a densitometric curve-based method that evaluates the intensity as well as the position of the bands to generate pairwise similarity scores (Pearson coefficient) that were used for cluster analysis. Cluster analysis was performed by constructing dendrograms using the unweighted pair-group method and the arithmetic mean method of tree-building. A similarity score of at least 85% was used as cut-off for designating clusters of similar isolates, where one band difference between patterns was allowed.

MLST was performed on all isolates according to [35], using internal fragments of the following seven house-keeping genes: *adk* (adenylate kinase), *fumC* (fumarate hydratase), *gyrB* (DNA gyrase), *icd* (isocitrate/isopropylmalate dehydrogenase), *mdh* (malate dehydrogenase), *purA* (adenylosuccinate dehydrogenase), and *recA* (ATP/GTP binding motif). Sequence types were assigned by submission of the sequences to the *E*. *coli* database (available at http://mlst.warwick.ac.uk/mlst/dbs/Ecoli).

The CTX-M genotype of all isolates was determined using a recently described, multiplex real-time -PCR specific for the 5 major groups of CTX-M enzymes [29]. Further identification of CTX-M genes was done by Sanger sequencing on a ABI3500 DNA analyzer of a PCR amplicon obtained with primers F:5’-GGGTGAAGTAAGTGACCAGAATCA-3’, and R:5’-CACGTCAATGGGACGATGTC-3’.

### Data analysis and statistics

All patient data and data obtained from AFLP cluster analysis, CTX-M PCR results and VITEK® 2 antimicrobial susceptibility tests were processed and analysed in Microsoft Excel version 2010. Additional statistics were calculated using Graphpad Prism version 5.01 (Graphpad Inc. La Jolla, CA, USA).

## Results

### Characteristics of MDR *E*. *coli* isolates

During the period between April and September 2013 a total of 2401 *E*. *coli* isolates was identified, including 280 isolates (12%) isolated from 222 patients that were classified as MDRO. Only the first isolate of each of 222 patients was included for analysis. One-hundred and fifty-seven of 222 isolates (71%) were confirmed to produce ESBL using disk diffusion, with or without additional resistance to aminoglycosides and quinolones. The other 65 isolates (29%) were resistant to aminoglycosides and quinolones but did not produce an ESBL.

### Genotyping results of MDR *E*. *coli*

All isolates were submitted to AFLP genotyping. Amongst the 222 isolates, 47 unique AFLP profiles were found, next to 35 clusters of 2 to 8 isolates and one large cluster of 50 isolates with similar profiles (Table 1). Additional MLST identified all isolates in the large cluster as the ESBL-associated widespread ST131. Some of the neighbouring clusters with related AFLP profile also contained ST131 isolates. In total 68 isolates (31%) were identified as ST131 (Table 2). Other sequence types that were found in the smaller clusters were ST10, 23, 38, 73, 155, and 405. Finally, in 129 out of the 222 isolates a CTX-M gene was found, of which 93 isolates (42%) contained CTX-M group I, which includes CTX-M 15 (Table 2). Most other CTX-M genes belonged to CTX-M group IV (13%), which includes CTX-M 14.

**Table 1:** AFLP genotyping results of MDR *E*. *coli* in patient groups.

Cluster size^1^	All isolates (n = 222)		Group I (n = 127)		Group II (n = 95)	
	no. of clusters	proportion of all isolates	no. of clusters	proportion of Group I isolates	no. of clusters	proportion of Group II isolates
1 (unique profile)	47	21,2%	44	34,6%	27	28,4%
2	13	11,7%	9	14,2%	13	27,4%
3	9	12,2%	4	9,4%	3	9,5%
4	2	3,6%	1	3,1%	3	12,6%
5	3	6,8%	3	11,8%	-	-
6	2	5,4%	-	-	-	-
7	3	9,4%	1	5,5%	-	-
8	2	7,2%	-	-	-	-
>10	1 (50)	22,5%	1 (27)	21,3%	1 (21)	22,1%
Total no. of different clusters	92	100,0%	63	100,0%	47	100,0%

^1^Number of isolates per cluster with identical AFLP profiles is indicated. Profiles with a similarity score of at least 85%, allowing maximally one different band (Fig 1), were considered as identical.

**Table 2:** Distribution of sequence types and CTX-M plasmids in MDR *E*. *coli*.

Genetic marker	All isolates (n = 222)	Group I (n = 127)	Group II (n = 95)
ST131 present	68 (31%)	40 (32%)	28 (30%)
CTX-M present, total	129 (58%)	71 (56%)	58 (61%)
CTX-M group I	93 (42%)	51 (40%)	42 (44%)
CTX-M group II	6 (3%)	0	6 (6%)
CTX-M group III + V	2 (1%)	1 (1%)	1 (1%)
CTX-M group IV	28 (13%)	19 (15%)	9 (10%)
no CTX-M group	93 (42%)	56 (44%)	37 (39%)

A similar, heterogeneous range of different AFLP profiles was found amongst isolates from both screening and non-screening cultures (data not shown). Notably, 45 out of the 68 isolates (66%) identified as ST131 were obtained from rectal screening cultures, indicating intestinal colonization by this clonal group within our patient population.

In general, antimicrobial resistance rates were similar between the two largest CTX-M groups, with the exception of a higher resistance rate to fluoroquinolones in CTX-M group I (75% vs 61%, respectively; S1 Table). Amongst ST131 isolates, resistance rates against third generation cephalosporins and fluoroquinolones were higher compared to non-ST131 isolates (76% vs 65% and 77% vs 64%, respectively; S2 Table).

### Definition and characteristics of patient groups with MDR *E*. *coli*

To compare genotyping results between patients that more likely carried community-associated isolates and patients that were more likely to have acquired a hospital-associated isolate, we divided all patients in two groups based on hospitalisation status and, in case of hospitalization, the number of days between admittance and strain isolation. Fifty-nine out of the 222 MDR *E*. *coli* positive patients (27%), mostly outpatients and nursing home residents, were not hospitalized at the time of strain isolation. Amongst the 163 hospitalized patients (73%) the number of days between hospital admission and isolation of the first MDR *E*. *coli* ranged from 0 to 86 days (median 6 days, interquartile range (IQR) 1–10 days). In total, 127 patients (57%) were either not hospitalized or hospitalized less than 72 hours at time of strain isolation (group I, community associated), while 95 patients (43%) were hospitalized at least 72 hours at time of the first strain isolation (group II, hospital associated).

None of the hospitalized patients in group II were known to have a culture positive for MDR *E*. *coli* in the last 6 months prior to hospital admission. Age and sex distribution were similar for both groups (Table 3), as was postal code distribution (data not shown). We did not observe a relative overrepresentation of any clinical ward amongst hospitalized patients carrying MDR *E*. *coli* (data not shown).

**Table 3:** Basic parameters of included patients.

Parameter	Total	Group I^1^	Group II^2^
No of patients/isolates, n	222	127	95
Median age (IQR)	66 (52–77)	62 (43–77)	69 (58–78)
Male sex, n (%)	131 (59%)	70 (55%)	57 (60%)
Median days until pos. culture (IQR)^3^	6 (1–10)	0 (0–1)	9 (5–11)
Median no. of past hospitalizations (IQR)^4^	0 (0–2)	0 (0–1)	1 (0–2)

^1^Group I includes isolates obtained from either non-hospitalized patients or from patients within 72 hours of hospital admission.

^2^Group II includes isolates obtained from patients after 72 hours of hospital admission.

^3^Days until positive culture was defined as the number of days between date of admittance and the date of strain isolation. Cases of no hospitalization or isolation at the date of admittance were defined as 0 days.

^4^The number of hospitalizations in the last year, excluding any current hospitalization, were documented. Hospitalization was defined as a stay of at least 24 hours in any hospital-associated location. Only data on hospitalizations in our hospital were available.

### Susceptibilities and genotyping results of MDR *E*. *coli* in the community associated patient group

Of the 127 MDR *E*. *coli* in the community-associated patient group I, antimicrobial susceptibility data were available for 117 isolates (Table 4). Of these 117 isolates, 75 isolates (64%) were resistant for ceftazidim and cefotaxim indicating ESBL production. ESBL production was confirmed with disk diffusion for 72 isolates. Plasmid encoded CTX-M enzymes were found in 69 ESBL positive- and in 2 phenotypically ESBL negative isolates. We did not detect a CTX-M enzyme in 3 isolates confirmed to produce ESBL, suggesting the presence of another β-lactamase for which we did not screen.

**Table 4:** Antimicrobial resistance rates of MDR *E*. *coli*.

Antibiotic class	Total of resistant isolates (n = 208)^1^	Resistant isolates group I (n = 117)	Resistant isolates group II (n = 91)
Any beta-lactam	204 (96%)	114 (97%)	90 (99%)
3d generation cephalosporins	142 (68%)	75 (64%)	67 (74%)
Quinolones	142 (68%)	88 (75%)	54 (59%)
Trimethoprim/ sulphonamides	115 (55%)	64 (55%)	50 (55%)
Aminoglycosides	96 (46%)	63 (54%)	33 (36%)

^1^ Antimicrobial susceptibility data was available for 208 out of the 222 isolates included in this study.

Sixty-three isolates (54%), of which 32 isolates produced ESBL were resistant to aminoglycosides, while 88 isolates (75%), of which 53 isolates produced ESBL, were resistant to quinolones (Table 4, S1 Table). Thus, ESBL production is not a prerequisite for resistance to non β-lactam antibiotic groups.

**Fig 1:**
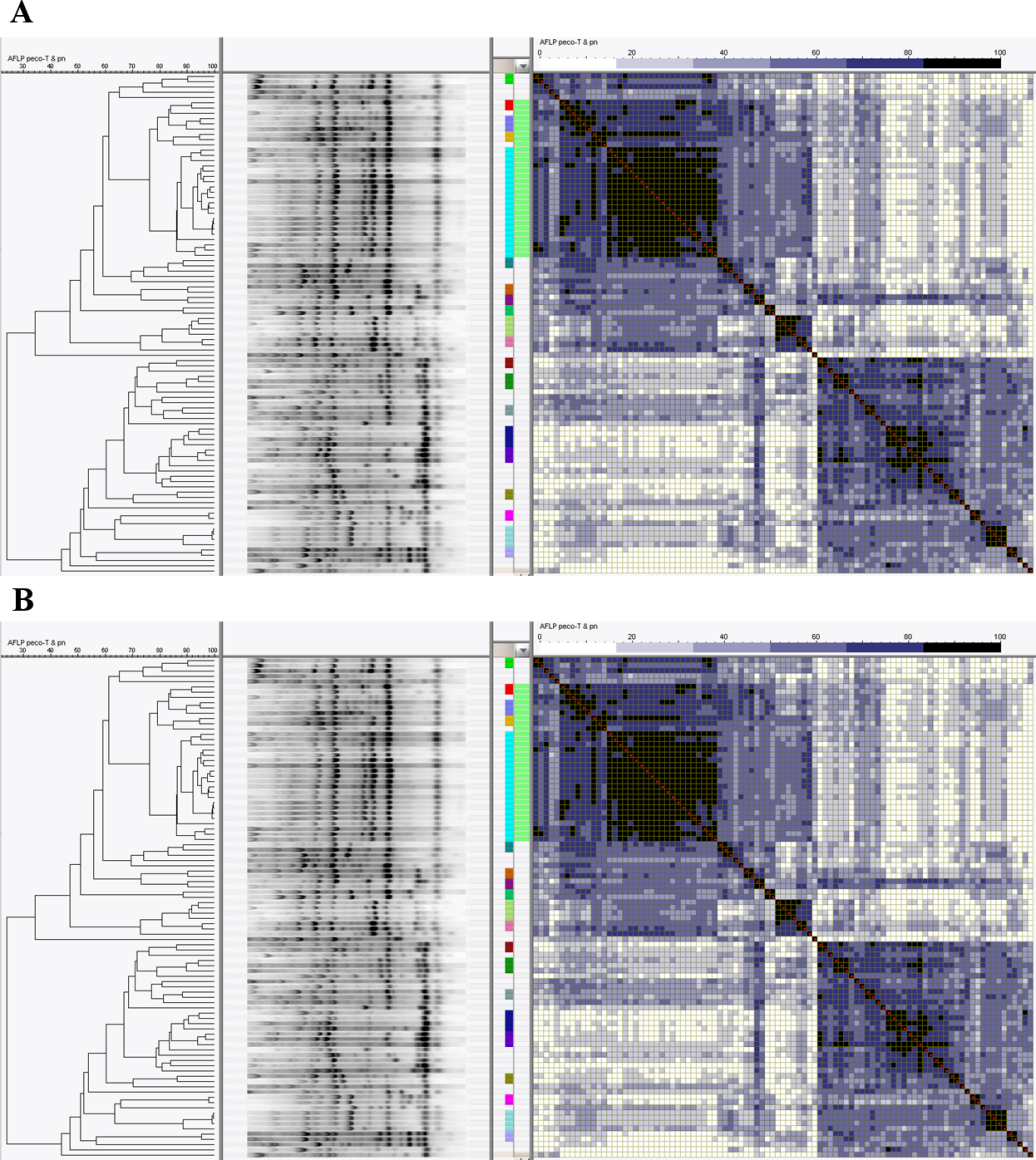
Genotyping of multi-drug resistant *E*. *coli*. Cluster analysis based on AFLP data with (**A)** similarity matrix of community associated isolates (group I) and (**B)** hospital associated isolates (group II). AFLP clusters are indicated by dark boxes, whites are unique profiles. The green bar adjacent to the similarity matrix indicates Sequence Type 131, color-coded boxes to the left of this indicate clusters with identical AFLP profiles.

AFLP genotyping of isolates in this patient group showed that 44 out of 127 isolates (35%) had an unique profile when >85% similarity was considered as a single strain type (Fig 1A, Table 1). The other 83 isolates (65%) grouped in 19 clusters with a range of 2–27 isolates per cluster. All 27 isolates within the largest cluster were identified as ST131, next to 7 and 2 other ST131 isolates amongst neighbouring clusters with related AFLP profiles. In total 40 ST131 isolates were identified in this patient group. CTX-M group I, which includes CTX-M15, was the most abundant amongst all isolates, being found in respectively 22 isolates (Table 2). Sixteen of these isolates were identified as ST131. Loss of plasmid would expectedly revert the susceptibility of 17 of 22 isolates to the 3d generation cephalosporins.

### Susceptibilities and genotyping results of MDR *E*. *coli* in the hospital associated patient group

Of the 95 MDR *E*. *coli* isolates in the community-associated patient group II, antimicrobial susceptibility data was available for 91 isolates. Sixty-seven (74%) of these isolates were resistant to ceftazidim and cefotaxim, indicative of ESBL production, which was confirmed for 66 isolates. Plasmid encoded CTX-M enzymes were shown present in 57 ESBL positive isolates and one ESBL negative isolate. We did not detect a CTX-M enzyme in 9 isolates with confirmed ESBL production.

Resistance to other antibiotic classes was lower in this patient group compared to the community-associated group I: 33 isolates (36%) were resistant to aminoglycosides, of which 16 were confirmed to produce ESBL, while 54 isolates (59%) were resistant to quinolones, of which 33 isolates were confirmed to produce ESBL (Table 4).

Genotyping of isolates in this patient group showed that 27 out of 95 isolates (28%) had an unique AFLP profile when >85% similarity was considered as a single strain type (Fig 1B, Table 1). The remaining 68 isolates (72%) were grouped in 20 clusters with a range of 2–21 isolates per cluster. All 21 isolates within the largest cluster were identified as ST131. Together with 7 ST131 isolates in neighbouring clusters, a total of 28 ST131 isolates was found in this patient group. The large ST131 cluster contained both isolates with and without beta-lactamase encoded by one of the CTX-M enzymes, with CTX-M15 being detected in respectively 14 isolates, and CTX-M22 in one isolate. Loss of plasmid would expectedly revert the susceptibility of 12 out of 14 isolates to antibiotic sensitive.

In 5 clusters within this patient group (median of 2 patients per cluster) identical AFLP profiles were isolated from different patients who shared the same ward at overlapping hospitalization periods. Although in 3 of these clusters identical profiles were also isolated from demographically unrelated patients, this suggests that limited transmission of MDR *E*. *coli* might occur within the hospital.

### Comparison of genotyping results between patient groups

The percentages of isolates in group I and II containing unique ALFP profiles were respectively 35% and 28%, and both patient groups contained one large, related AFLP cluster comprising 27 and 21 isolates, respectively (Table 1). All isolates within these large AFLP clusters were part of the larger ST131 clade. Almost all other AFLP clusters in both patient groups were unrelated. The relative number of identified ST131 isolates was similar in both patient groups with respectively 32% and 30% (Table 2). Finally, CTX-M group I plasmids, including CTX-M 15, were the most abundant in both patient groups with 40 and 44% of isolates containing this plasmid, respectively. Altogether, these data indicate a similar distribution of genetic markers in both patient groups.

## Discussion

Our aims were to investigate the extent and direction of clonal spread of MDR *E*. *coli* amongst the heterogeneous patient population within our hospital. Genotyping showed that most multi-drug resistant *E*. *coli* either had unique AFLP profiles or grouped in small clusters of maximally 8 isolates. We identified one large ST131 clade comprising 33% of all isolates, which contained several AFLP clusters with similar profiles.

The large AFLP clusters in both patient groups were identical in their AFLP profiles while other clusters (but one) differed between patient groups, reflecting the high genetic diversity of *E*. *coli*. The genetic heterogeneity of multi-drug resistant *E*. *coli* found in this study, isolated both in hospitals and the community, is in line with an abundance of previous studies [6, 36–41]. This heterogeneity contrasts with other multi-drug resistant organisms that are mostly dominated by specific hospital-associated clones [20, 21, 26, 27].

Although genetic heterogeneity was slightly higher in the community-associated patient group (35% vs. 28% of isolates containing unique ALFP profiles in group I and II, respectively), the distribution of CTX-M plasmids was similar in both groups with CTX-M group I, including the endemic community-associated CTX-M 15 [5, 6, 38], being the most prevalent (40% vs. 44% of isolates in group I and II, respectively). These findings are in line with other recent data demonstrating similar distributions of CTX-M plasmids between community- and hospital-onset infections with ESBL *E*. *coli* [42]. In addition, the distribution of the CTX-M-15 associated ST131 lineage was also similar in both patient groups. Altogether, these results suggest that MDR *E*. *coli* are mostly acquired in the community and are not replaced by hospital-associated clones in hospitalized patients.

The large, community-associated cluster of mainly ST131 *E*. *coli* identified in this study further implicates the emergence of this clonal group amongst clinical isolates [8]. Emergence of particular clonal lineages has recently also been observed for multi-drug resistant *Klebsiella pneumoniae* [26] and *E*. *coli* in a specific patient population under antibiotic pressure [43]. Alternatively, the large cluster that we identified might reflect the distribution of genetic profiles of *E*. *coli* within our local population. The proportions of clusters and unique banding patterns that we found appears to be similar to those found for *E*. *coli* in the general environment [36].

Considering the high discriminatory power of AFLP [30, 31] and confirmation of results with CTX-M group analysis and MLST, we did not include additional molecular typing such as detailed sequence analysis of CTX-M, fimH, gyrA or parC in this study for further confirmation. In fact, AFLP analysis has a very high concordance with phylogenetic assignation e.g. based on gyrA sequence typing [30]. Nonetheless, additional typing will be an interesting addition to future studies to further explore the distribution of particular subclones of *E*. *coli* within patient groups, such as the H30 subclone of ST131, and their association with particular antimicrobial resistance patterns [44].

In the current study, the included screening cultures mainly consisted of rectal swabs, obtained from a pre-defined selection of patients within our hospital. Although other patient characteristics such as underlying disease and hospital ward of admission were heterogeneous, we cannot rule out that the above mentioned selection might bias the distribution of genetic markers found amongst isolates. However, AFLP profiles of the 38 non-screening cultures, all from extra-intestinal origin, did not form a separate cluster but randomly clustered together with *E*. *coli* from intestinal origin. Therefore, we currently do not have an indication of bias within our data. Nonetheless, future studies should non-selectively screen for MDR *E*.*coli* in all patients at multiple anatomical sites (e.g intestinal and urine) over a longer period.

The cut-off of 72 hours to define community- and hospital-associated patient groups was chosen in line with other recent studies [3, 17] as not to overestimate the number of hospital-acquired infections [18, 34]. This cut-off remains somewhat arbitrary as we did not screen all patients at admission for MDR *E*. *coli*. Therefore, we cannot say whether isolates are truly community- or hospital acquired. Yet in a small subset of 24 patients in the hospital-associated group with earlier negative screening cultures for MDR *E*. *coli* during the same admission suggesting true hospital acquired isolates, separate genotyping identified 12 unique profiles (50%) and 6 clusters of 2 isolates (50%) (data not shown). These findings further support our results suggesting that hospitalized patients do not acquire a specific hospital-associated clone.

Within our hospital, isolated MDROs are routinely genotyped to enable recognition of potential transmission among patients. We observed limited transmission of identical AFLP profiles of MDR *E*. *coli* between hospitalized patients sharing the same ward at overlapping hospitalization periods. However, we did not fully sequence CTX-M plasmids and therefore cannot confirm whether transmission of plasmids occurred between isolates of different patients. Yet in the absence of a clear hospital-associated outbreak situation and given our results that most MDR *E*. *coli* are likely acquired in the community, it remains debatable whether this required labour for monitoring is justified in the daily diagnostics laboratory.

Although for many *Enterobacteriacea* routine genotyping has proven to be very useful, in view of our findings the monitoring of potentially emerging, clinically significant clones of MDR *E*.*coli* seems less meaningful. Further studies including routine screening of all patients at admittance, more detailed documentation of demographics and sequencing of plasmids are required to determine the true transmission rate and effectiveness of screening and transmission control for MDR *E*. *coli*.

## Supporting Information

S1 Table Antimicrobial resistance rates of MDR *E*. *coli* according to CTX-M group. (DOCX)

S2 Table Antimicrobial resistance rates of MDR *E*. *coli* according to ST131 group. (DOCX)
