# Supplementary material for: The Widespread Presence of a Multidrug-Resistant Escherichia coli ST131 Clade among Community-Associated and Hospitalized Patients
Source: PLoS One. 2016 Mar 1;11(3):e0150420. doi: 10.1371/journal.pone.0150420 (PMC4773163; doi:10.1371/journal.pone.0150420)
Supplement: S1 Table — (DOCX) [file pone.0150420.s001.docx]

**S1 Table. Antimicrobial resistance rates of MDR *E. coli* according to CTX-M group.**

|  | Total (n=208)^1^ | CTX-M group I (n=93) | CTX-M group II (n=6) | CTX-M group III+V (n=2) | CTX-M group IV (n=28) |
| --- | --- | --- | --- | --- | --- |
| Any beta-lactam | 204 (96%) | 92 (99%) | 6 (100%) | 2 (100%) | 27 (96%) |
| 3d generation cephalosporin | 142 (68%) | 90 (97%) | 6 (100%) | 2 (100%) | 26 (93%) |
| Quinolones | 142 (68%) | 70 (75%) | 1 (17%) | 0 (0%) | 17 (61%) |
| Trimethoprim/ sulphonamides | 115 (55%) | 58 (62%) | 6 (100%) | 1 (50%) | 16 (57%) |
| Aminoglycosides | 96 (46%) | 40 (43%) | 0 (0%) | 0 (0%) | 12 (43%) |

^1^ Antimicrobial susceptibility data was available for 208 out of the 222 isolates included in this study.
