# Supplementary material for: The Widespread Presence of a Multidrug-Resistant Escherichia coli ST131 Clade among Community-Associated and Hospitalized Patients
Source: PLoS One. 2016 Mar 1;11(3):e0150420. doi: 10.1371/journal.pone.0150420 (PMC4773163; doi:10.1371/journal.pone.0150420)
Supplement: S2 Table — (DOCX) [file pone.0150420.s002.docx]

**S2 Table. Antimicrobial resistance rates of MDR *E. coli* according to ST131 group.**

|  | Total (n=208)^1^ |  | ST131 (n=66)^1^ | Non-ST131 (n=142)^1^ |
| --- | --- | --- | --- | --- |
| Any beta-lactam | 204 (96%) |  | 67 (99%) | 137 (96%) |
| 3d generation cephalosporin | 142 (68%) |  | 50 (76%) | 92 (65%) |
| Quinolones | 142 (68%) |  | 51 (77%) | 91 (64%) |
| Trimethoprim/ sulphonamides | 115 (55%) |  | 35 (53%) | 80 (56%) |
| Aminoglycosides | 96 (46%) |  | 32 (48%) | 64 (45%) |

^1^ Number of isolates per group are indicated for which antimicrobial susceptibility data was available.
